# Geographic Disparities of Comorbidities in Mortality of Patients With Alzheimer’s Disease

**DOI:** 10.1093/geroni/igaa057.3125

**Published:** 2020-12-16

**Authors:** Julia Kravchenko

**Affiliations:** Duke University, Durham, North Carolina, United States

## Abstract

Comorbidities can contribute to the gap in Alzheimer’s disease (AD) mortality between the East and West coast U.S. Using Multiple-Cause-of-Death and 5%-Medicare data, we analyzed age-adjusted (65+) mortality rates from AD in two Health and Human Services (HHS) areas with opposed mortality patterns in 2010-2018: 150.9±0.6/100,000 in HHS2 (NJ,NY) and 363.1±1.5/100,000 in HHS10 (AK,ID,OR,WA). Co-existing diabetes, heart failure, cerebrovascular, digestive, and kidney diseases significantly contributed to this gap, while contribution of heart diseases reduced its magnitude. An unexpectedly strong effect (higher rate in HH10 by a factor of 3-5) was identified for symptoms/signs that are not from identified specific diseases, life-threatening injures/falls and other external causes that are common among patients with AD. We concluded that although contributions of comorbidities with well-developed treatment guidelines (e.g., heart disease) to geographic disparities in AD mortality were small, the disparities can be generated by unexpected comorbidities including diseases with poorly defined conditions.

